# Development of a Pavement-Embedded Piezoelectric Harvester in a Real Traffic Environment

**DOI:** 10.3390/s23094238

**Published:** 2023-04-24

**Authors:** Lucas Fraporti Heller, Lélio Antônio Teixeira Brito, Marcos Antônio Jeremias Coelho, Valner Brusamarello, Washington Peres Nuñez

**Affiliations:** 1 Pavement Laboratory—LAPAV, Universidade Federal do Rio Grande do Sul—UFRGS, Porto Alegre 90040-060, Brazil; 2 Industrial Systems Laboratory—LSI, Universidade Federal do Rio Grande do Sul—UFRGS, Porto Alegre 90040-060, Brazil

**Keywords:** energy harvesting, piezoelectric transducer, road pavements

## Abstract

Road pavements are spread over large areas and convey various possibilities for energy sources such as high thermal gradients due to their materials and colors, wind corridors, large flat areas for solar harvesting, and heavy loading from traffic. The latest advances in road energy generation have been discretely implemented and have mainly focused on photovoltaic surface applications; other studies have explored the use of piezoelectric transducers with high stresses for better energy-production performance but limited life span. This study explores the stresses on pavement surfaces from traffic loading shockwaves that yield to the natural frequency vibration a piezoelectric harvester using a cantilever array. The passing vehicles triggered 16 piezoelectric sensors divided into four embedded steel profiles. The peak electrical power obtained in the experiment was 55.6 µW with a single transducer using a tip mass of 16 g. The proposed harvester demonstrated potential for applications in micro-generation of energy with limited infrastructure modification and high endurance under traffic loading over time. Its generation capacity is around 50 mWh a month with 16 piezoelectric cantilevers installed (for a commercial traffic volume of 1500 vehicles a day), enough to power a 200 m flashing LED raised marker strip to guide drivers for lane alignment during night shifts.

## 1. Introduction

A large amount of energy is wasted during the operation and construction of road transport. Different methods are studied to mitigate the environmental damage of this mode of transport to obtain a more sustainable infrastructure. Among these methods is the use of the energy harvesting (EH) concept to collect the wasted thermal and kinetic energy of road and highway environments, allowing renewable energy generation.

The amount of energy generated by pavement EH systems is usually small and has steep prices compared with conventional energy sources. However, it earns interest for facilitating the installation of lighting and monitoring systems and data collection in remote zones, allowing a power grid-free system. The usual mechanisms for EH in research on in-road applications are piezoelectric transducers, electromagnetic generators, thermoelectric generators, photovoltaic panels, and solar collectors [1].

The possibility of using paved areas for power generation matches the current focus of civil engineering on creating more sustainable infrastructure. Early models that take advantage of thermal energy in paved areas included the use of fluid-filled ducts embedded in the pavement to control the surface temperature, allowing snow melting, temperature reduction, and air conditioning in nearby buildings [2]. This method uses thermal energy from solar radiation and water as the fluid.

Alternatives include using air-filled ducts instead [3,4,5]. When the air is heated, convection causes hot air to circulate through the duct and chimney system, passing through a turbine that generates electric power. The advantages of this system are a lower risk of leakage in the pavement structure and a reduced pavement temperature, mitigating urban heat island effects.

Pavement heat can also be collected with thermoelectric cells using the Seebeck effect, which involves the generation of electricity from a temperature differential between the cold and hot sides of the cell [6,7,8,9]. The use of thermoelectric generators takes advantage of the high surface temperatures in combination with the lower temperatures of base layers. This effect is generally amplified using heat sinks and radiators, allowing generators to receive the maximum thermal amplitude on their sides.

Photovoltaic systems have experimental applications performed at roads in different locations. One successful experiment is the SolaRoad bike path in Krommenie, the Netherlands, although loads from bikes are very low compared with cars and trucks. Roadway use has been tested in France to evaluate the use of road photovoltaic panels. However, several problems have been reported [10] in the application of this highway, such as excessive noise, rapid degradation, and less than expected electricity generation, indicating that today’s state-of-the-art panels face a problem in withstanding heavy traffic.

In addition to thermal energy, pavements receive a large amount of kinetic energy from vehicles. This energy can be harnessed using electromagnetic generators or piezoelectric transducers. Electromagnetic generators generate energy through a coil and a magnet. Electromagnetic induction occurs when the system suffers displacement from traffic or pedestrians [11,12,13]. These generators have a good electrical output; however, the system is not level with the pavement, which makes it difficult to install on high-speed highways and streets but better suited for speed bumps.

Piezoelectric materials can generate electrical energy when subjected to stress and show a reverse effect whereby they undergo deformation when subjected to an electric field. Different materials have piezoelectric characteristics, but the most common for applications is the piezoceramic lead zirconium titanate (PZT) [1].

This material features a high electric conversion but is extremely rigid and brittle [1]; therefore, they are predisposed to fatigue over time [14]. Piezoelectric energy harvesters (PEHs) can be installed in pavements using different geometries and ways to apply stress, such as direct compressive strain on PZT piles and disks [15,16,17,18,19,20], cymbal forms [21,22,23], bridges [24,25,26,27], cantilevers [28], and beams fixed at both ends [29]. Another possibility presented is the use of flexible piezoelectric materials, such as poly(vinylidene fluoride) (PVDF) [14] transducers, which avoid fatigue and cracking problems [30].

The cymbal forms consist of a piezoelectric disc with two metal layers. This format was studied and reached a generation power of 1.20 mW considering discs 32 m in diameter and applied loads with a frequency of 20 Hz [21]. An experiment using cymbal transducers achieved a maximum power output of 16 µW per transducer. An energy density of 5.18 kWh/m^2^ would be achieved with 30,000 cymbals installed in 100 m of pavement [23].

It is worth pointing out that the use of piezoelectric transducers on highways generally requires protection for the materials and cables. For this purpose, a protective case is usually developed, allowing the transducers to be embedded in the pavement. A prototype developed using piezoelectric disks and protection was installed on a commercial scale. The authors achieved an instantaneous maximum power of 3.106 W [17].

In a recent experiment, a prototype with 80 piezoelectric beams with both ends fixed and a small displacement at the center of the transducers achieved an instant power of 2.08 W [29]. These results show a good potential for small applications of piezoelectric energy harvesters for traffic sensors and indicators.

A possibility for PEHs to avoid direct application of stress in the transducers is the use of systems based on vibration from traffic. This prototype to be inserted in the floor uses cantilevers with tip masses [28]. Using only vibration should avoid excessive stress in the PZT, avoiding cracks and fatigue. The tip masses aim to optimize the generated power according to the load and speed, allowing greater power generation during loading applications. According to the authors, the prototype with 12 transducers is able to generate 184 µW of electrical power.

Fatigue of the PZT elements is well-known [31,32,33,34] and it is important to achieve an energy harvester that can withstand the loads during the pavement life cycle. Research by Mizuno et al. [30] indicates the likelihood of PZT failure with a number of cycles equal to 1 to 106, depending on the compressive stress and geometry of the elements. Considering a road segment with 1000 vehicles a day (with a minimum of two axles per vehicle) of mixed traffic, more than 70% of the total number of cycles to failure would be reached in one year.

To avoid the direct stress and fatigue on the PZT transducers, the use of cantilevers and vibration-based systems is possible. These systems are used in other types of applications such actuators and check valves [35,36] and different fields, such as Internet of Things (IoT), Biomedicine, industrial devices, and more [37,38,39]. For use in roadways, it is necessary to study more applications [40].

### Objectives

The present work presents the deployment and preliminary results of a PEH prototype installed in an in-service highway in the Rio Grande do Sul State, Brazil, based on free vibration of the transducers. The system was installed in a toll plaza and supplied energy to flash a cat’s eye raised marker system. The main objective of the application is to evaluate the field behavior, resistance, and electrical output of a small-scale (four boxes totaling 16 transducers) harvester in an in-service highway.

A fundamental aspect of the study was to seek durability of the prototype for virtually infinite energy harvesting from natural pavement vibration due to a theoretically fatigue-free transducer setup. Since maintenance of a highway is undesirable, ideally the system should remain functional during the life cycle of the pavement structure.

This paper provides results obtained after the installation within a few weeks of the experimental work. It is expected that the proposed harvester can provide data regarding durability and required workarounds to tune the system in the first Brazilian application of the technology and, allegedly, one of the first applications of PEH on in-service roads the literature review can cover to date.

## 2. Materials and Methods

The long-term objective of using the generator to supply traffic equipment and data collectors was the primary reason for the choice of the piezoelectric system. Piezoelectric transducers feature traffic-dependent generation, climate independence, and easy handling for road installation. These features make the system theoretically compatible with the intended applications.

The transducers selected were PZT piezoelectric cantilevers working through vibration from the passage of vehicles over the system. The transducers were the commercially available PPA-1014 from MIDE, using the piezoceramics PZT-5H with an area of 500 mm^2^, with a sensitivity of 56 mV/g, capacitance of 24 nF, and a full-scale voltage range of 500 V.

The cantilever geometry was chosen to attenuate direct stress on the transducers, avoiding durability problems, excessive stress that can lead to fatigue, and cracking and breaking of the PZT elements.

The tests of the proposed generator were performed on a highway in service in the city of Gravataí in southern Brazil. The installation took place at the toll plaza located on the BR-290 Federal Highway. The toll plaza’s automatic lane received the systems so that vehicles would not stop over the boxes, maintaining a higher speed and providing greater generation. The installation of prototypes in the toll plaza allowed traffic information control during the experimental period.

**Figure 1:**
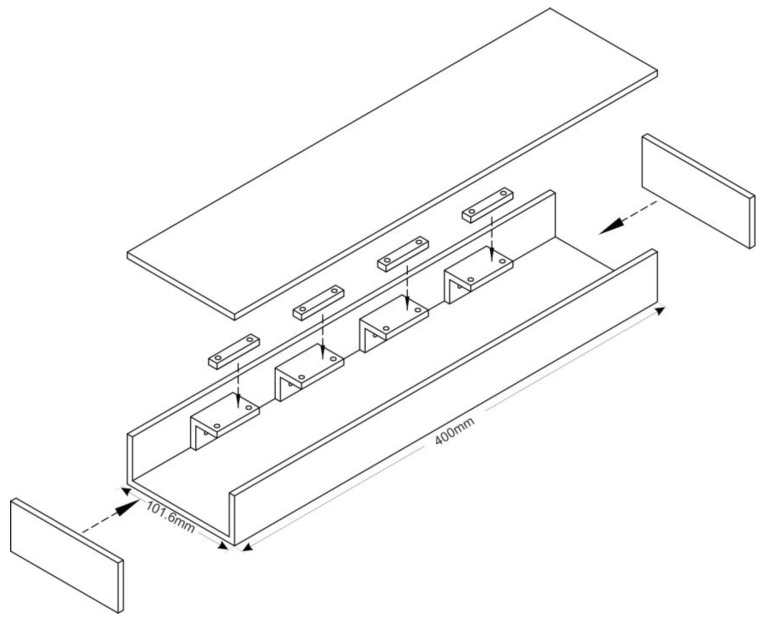
Dimensions of prototype box.

**Figure 2:**
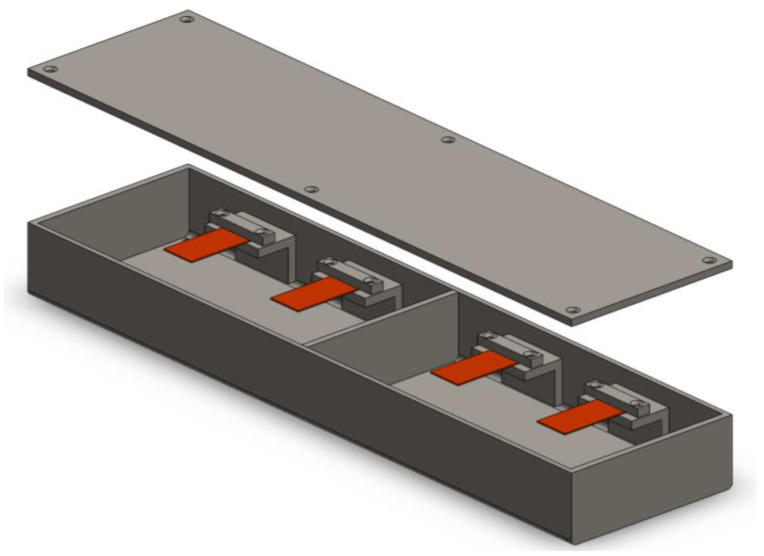
Schematic of prototype.

A case prototype was developed to ensure the safety of the piezoelectric transducers and allow room for system vibration and cables. The prototype body was designed using a structural steel beam segment with dimensions of 400 × 100 × 50 mm (length, width, height, respectively) and 4 mm thick walls (Figure 1 and Figure 2).

**Figure 3:**
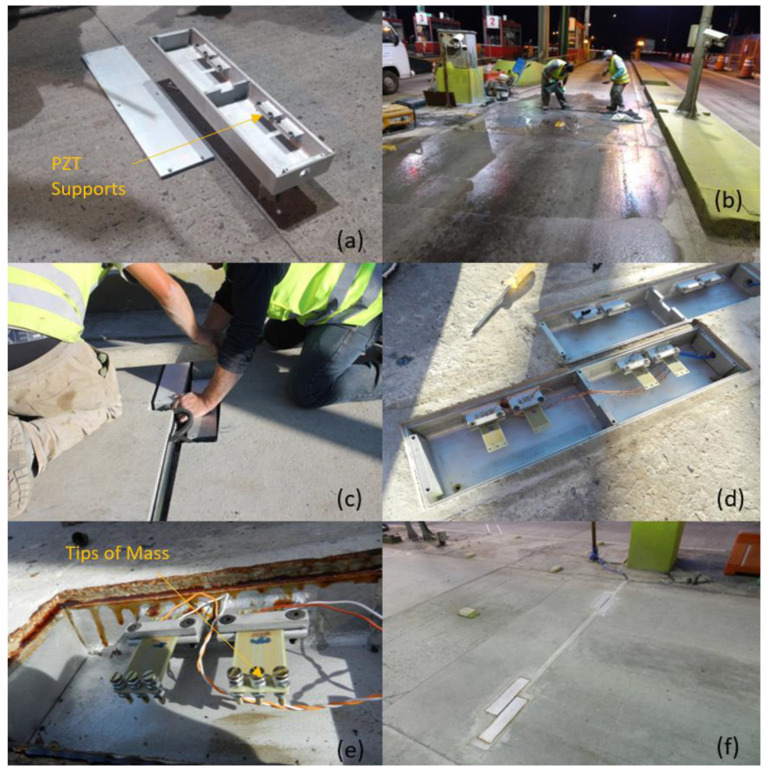
(**a**) Prototype box. (**b**) Preparation to install the prototypes in the automatic toll lane. (**c**) Prototypes leveled with the pavement. (**d**) Prototypes concreted in the pavement with transducers. (**e**) Transducers with tip masses. (**f**) Final results with the top cover sealed.

A total of four prototypes were built. The metal bodies had welded sides and a removable top cover for access to the components during operation. Internally, four cantilevers were placed in each box (Figure 3a), providing 16 transducers in total. The boxes were leveled with the concrete pavement surface (Figure 3b,c). The top cover was sealed with screws and joint adhesive, preventing water ingress (Figure 3d,f). Between the boxes, an elastomeric joint seal was applied to prevent movement that would impact vehicle traffic and can generate cracks in the pavement.

To evaluate generation, two different tip mass configurations were tested during the use of the system. First, the system used 6.7 g mass points on the transducers (Figure 3e). Subsequently, these tip masses were replaced by larger masses of approximately 16 g. Increasing the mass causes a greater amplitude of deformation during vibration, reaching higher generation potentials. However, larger deformation can damage the transducers, so smaller tip masses are applied first.

**Figure 4:**
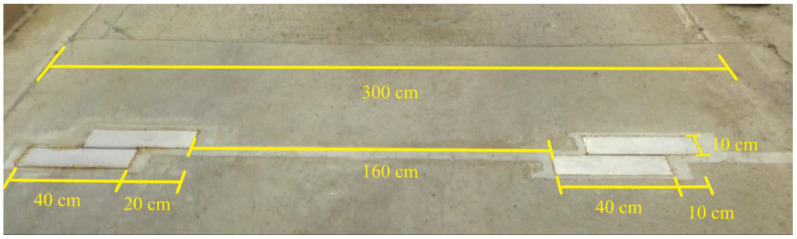
Positioning of the prototype in the lane (four boxes across the lane section for optimization of the wheel path coverage).

Two boxes were positioned on either side of the track to provide contact with both axles of the trucks. They were placed 80 cm from the center, based on the average truck axle width. The two boxes on each side had a slight gap between them, thus increasing the contact area (as seen in Figure 4). The prototypes were named from 1 to 4 from the right side of the track.

To evaluate the generation capability of the proposed system, a datalogger with a sampling rate of 1000 Hz was installed. The traffic data were recorded by the automatic toll lane where the prototypes were installed. A supercapacitor (12 F) was used to store the generated energy and a parallel 100 kΩ resistor was used to estimate the instant power output of the system. Then, the prototype was used to flash a twenty LED-powered cat’s eye system to beacon drivers along the lane using a stroboscopic effect.

## 3. Results

The system remained in operation for one month. The reading of voltage magnitude data from the transducers can also be used to identify different types of vehicles.

The time series of the output voltage signal demonstrating the operation of the system is described in Table 1 and can be seen in Figure 4. A small delay occurs due to the distance between the toll sensors and the PEH. As we can observe, different types of vehicles caused different output voltages. Heavy vehicles caused higher peak loads on the system. In addition, output differences between boxes happened because they were fixed at different positions on the road.

**Figure 5:**
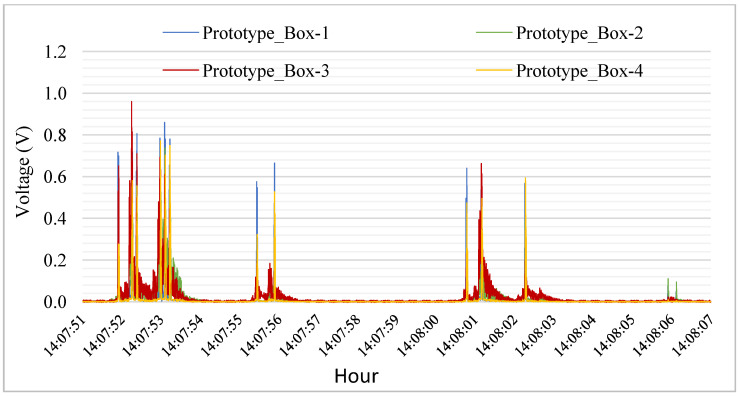
Electrical output caused by the vehicles.

**Figure 6:**
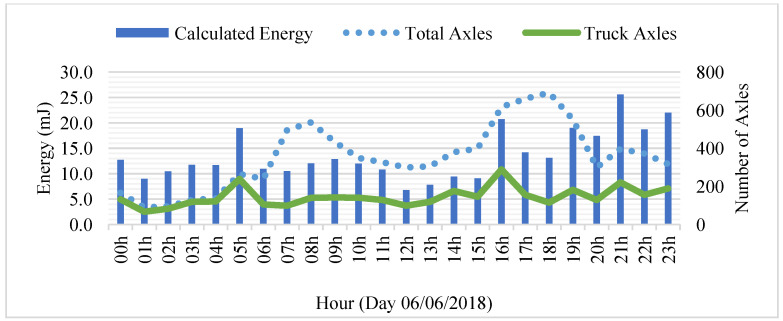
Results of a day of calculated energy and passing axles.

The dependence between the PEH and traffic was noted, analyzing a day of electrical output and the traffic during the same period. Individual analysis of Figure 5 showed that truck axles cause the higher peaks. During the day, the system has its higher peaks of output when the volume of truck axles is higher (as seen in Figure 6). Moreover, the heavier loads of truck axles, the positioning of the boxes, aimed at a greater axle width, is a factor to consider.

**Figure 7:**
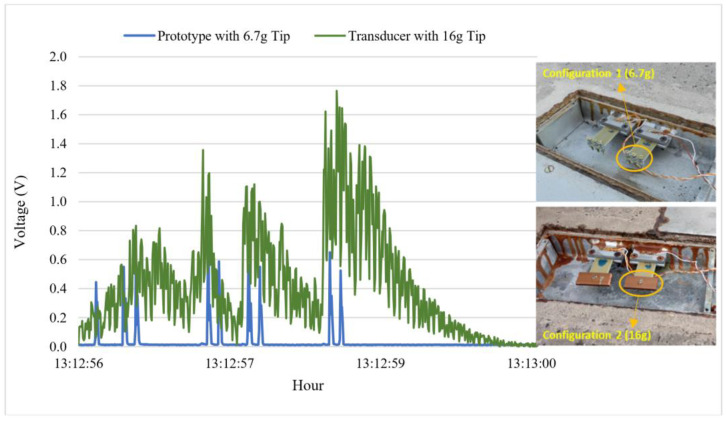
Comparison between tip masses connected to a resistor (100 kΩ).

To evaluate the use of heavier tip masses, the system was modified during the experiment. The great variety of traffic on the road made it difficult to obtain an optimal weight with simulations. Heavier tip masses were installed in prototype 1. The use of larger tip masses resulted in higher energy generation by the transducers. A comparison between the boxes with the highest tip masses shows that the system had a higher vibration after the initial impact of the vehicle axle. Figure 7 shows the difference in behavior between a transducer with a heavier tip mass and a prototype using a 6.7 g tip mass while connected to a 100 kΩ resistor. The subsequent vibration was noted during the passage of a nine-axle truck in the system.

**Figure 8:**
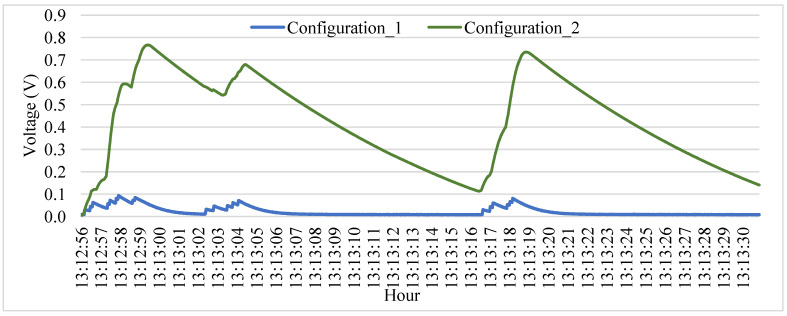
Difference between transducers with different tip masses connected to a capacitor.

Two transducers with different tips of mass were compared when connected to a small capacitor (22 µF) in prototype 1. Sensor 2 connected with the heavier tip mass reached almost 0.8 V during the passage of a truck, while sensor 1 achieved 0.1 V, as seen in Figure 8. As sensor 2 reached higher peaks during vehicle passage, it is possible to observe that the peaks end up accumulating during the passage of subsequent axles and vehicles, which contributes to a higher calculated energy.

The power output of the system was 19.2 µW for a prototype with four transducers. Using the heavier tip masses, the power achieved was 55.6 µW in a single transducer. The tip masses increase electrical generation by causing a greater deformation in the transducer beams. Additional care is necessary as they tend to shorten the fatigue life of the material. Further, increased tip masses decrease the vibration frequency of piezoelectric transducers. This lower frequency can cause interference with high frequencies of load application, thus decreasing the generation potential for high speeds.

### Truck Classification

The observed relationship between the peaks of electrical voltage and the passing axle on the highway opens the possibility of using piezoelectric transducers as traffic sensors. Such use is of great contribution to road management and deserves attention. The traffic data received indicates the categories of vehicles, according to the number of axles, since it is the information used by the toll plaza to charge the vehicles.

**Figure 9:**
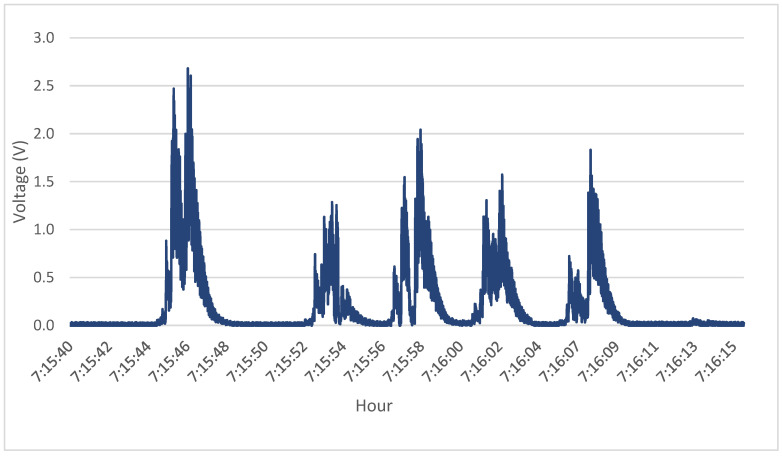
Voltage output for various commercial vehicles.

The heavier tip of mass on the piezoelectric transducer was responsible for a higher energy generation, as demonstrated earlier; however, the agitation of the transducer increased, generating secondary peaks that overlap with the main peaks of other axles. This behavior is demonstrated in the graph of Figure 9, showing a sequence of commercial vehicle passages. It is possible to relate the curves to the vehicle passages; however, they are not clear enough to identify the number of axles of each vehicle.

**Figure 10:**
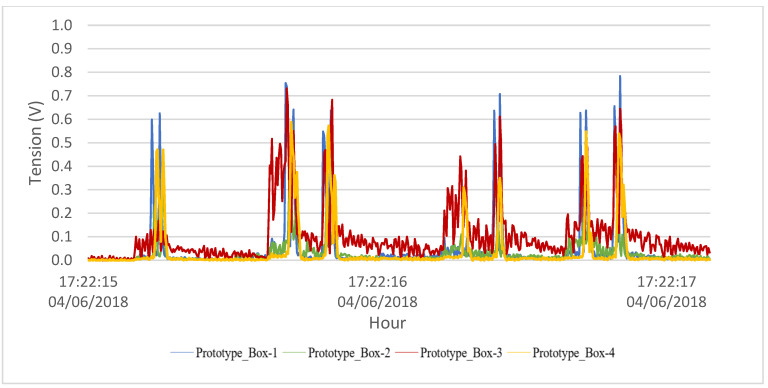
Voltage output of a single commercial vehicle with smaller tip of mass.

The use of a smaller tip of mass also generated vibration, but with less intensity, allowing for better identification of the vehicle axles. The generation curves of configuration A showed clear peaks coinciding with the number of axles, enabling the identification of the vehicle category through the data recorded by the system. This relationship is visualized in Figure 10, where a seven-axle vehicle is demonstrated in the system. The vibration of this vehicle was higher than in previous examples, but it is possible to distinguish the seven axles.

**Figure 11:**
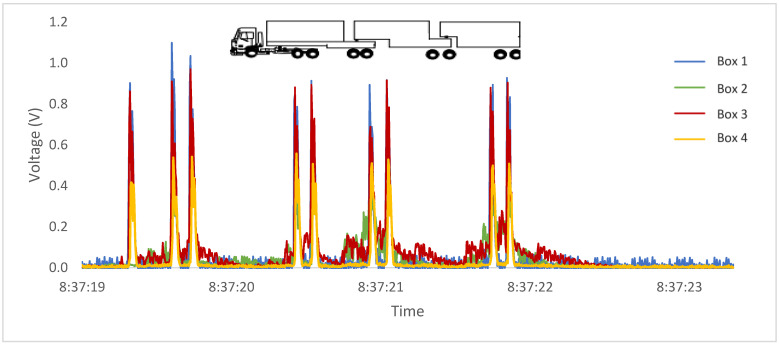
Passage of a 9-axle commercial vehicle with four tandem axles.

**Figure 12:**
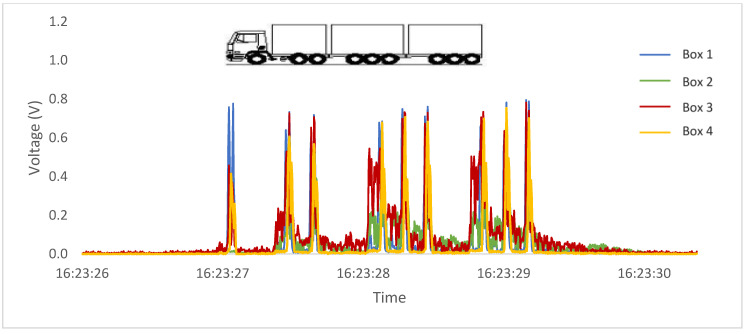
Passage of a 9-axle commercial vehicle with three tridem axles.

In addition to the possibility of axle counting, Figure 11 and Figure 12 show readings taken from two vehicles identified by the concessionaire as category 13 (9 axles) at two different times. The analyses have the necessary clarity to distinguish between different axle groups, demonstrating that both vehicles belong to two different categories according to the Brazilian classification, despite having the same number of axles.

**Figure 13:**
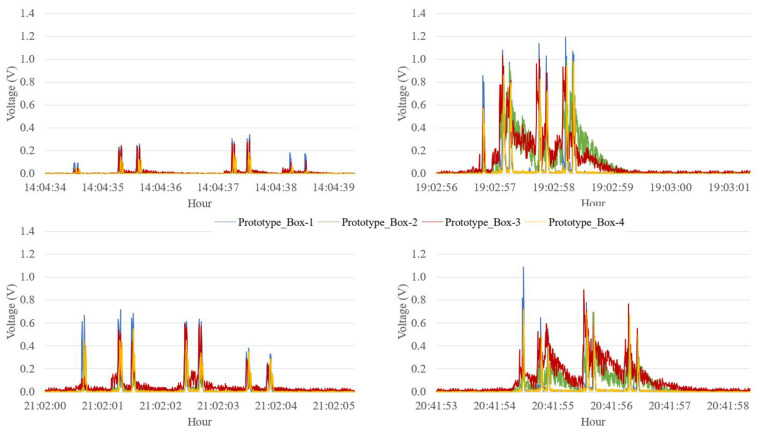
Different passages of 7-axle commercial vehicle.

Another situation of behavior alteration is shown in Figure 13, where vehicles with the same configuration and number of axles pass through the system at different times of the same day. The peaks generated in the system are similar; however, there is a significant difference in magnitude.

The graphs demonstrate the difference in magnitudes of the axles, ranging from 0.4 to 1.20 V in voltage peaks. It is also noted that the vehicle with lower amplitudes and vibration is more spread out in time, suggesting that its speed is slower than the other vehicles. Other behaviors can also be highlighted, such as the variation between the axle with the highest magnitude in each vehicle, indicating the possible distribution of load.

The speed of the vehicle and the load on each of its axles alter the system’s generation, as demonstrated in laboratory tests. Based on this behavior, it would be possible to perform field calibrations, relating the electrical voltages, speeds, and applied loads.

The system’s speed can be calculated by the difference between the electrical voltage peaks if the distance between the axles are known; however, since the spacing between axles varies, performing this check becomes difficult in the applied system configuration. An alternative would be to use two generators spaced in the direction of traffic, allowing the speed to be calculated by the time elapsed between the vehicle records on each generator.

Knowing the speed and electrical response, the load on the axles can be obtained through system calibrations and alterations to reduce unknown factors. The geometry used in this experiment can be optimized for generation, classification, or both purposes.

It is worth noting that the results in this experiment were obtained at speeds close to 40 km/h, the maximum allowed on the automatic toll road. Using this system at higher speeds may cause a differentiation of the electrical response curves for the tips of mass used. Due to the relationship between tips of mass and the loading speed, a study for the most appropriate mass is necessary for the application.

## 4. Conclusions

The small scale PEH was able to support traffic and weather during the experiment without displaying any evident signs of deterioration. A site inspection after 11 months evidenced no severe deterioration of the boxes, suggesting a long service life for the proposed harvester. The transducers submitted only to vibration should have a higher fatigue endurance, contributing to a long-term harvester.

The energy output is very low but high durability was demonstrated by the prototype box as well as the fatigue-free transducers. The spreading of installation costs over time, along with the low operational cost, means that the total energy generation also has a rather low cost. Moreover, generators can stack up many transducers in a small area, achieving a reasonably high energy density. The possibilities of energy generation away from the power grid would facilitate the installation of traffic data collection systems or lighting. Moreover, using an embedded system protects the harvesters from external damages, such as climate or vandalism.

The increase using a heavier mass in the cantilever was achieved due to large deformations in the tip of mass. Caution is recommended when using even heavier mass since it can reduce fatigue life of the PZT.

The low influence of passenger cars in generation indicates that modifications in the geometry to magnify the vibration to reach the transducers would allow a higher electrical output. These modifications should be carefully evaluated to maintain the resistance to heavy traffic of a highway.

For applications focusing on generation, the system should be installed in roads with high volumes of traffic. In this experiment, the application in a high-speed paying lane in a toll plaza allowed great control and easy access, but total traffic from the highway was divided between other paying lanes, reducing the energy harvesting potential. Heavy loads and high speed should also contribute to the generation potential.

Regarding the objective of using the harvester as a power source for data collectors, a large-scale and optimized design is necessary since the power output is low. However, the design showed potential to be a self-powered sensor, allowing axle counting and possibly load and speed estimation with further calibrations. Its capacity of generation is around 50 mWh per month with 16 piezoelectric cantilevers installed for a commercial traffic volume of 1500 vehicles a day, enough to power a 200 m flashing LED raised marker strip to guide drivers for lane alignment during night shifts.

## Figures and Tables

**Table 1:** Vehicles passing the PEH.

Hour	Vehicle Number	Type
04-06-2018 14:08:07	1	Six-axle truck
04-06-2018 14:08:11	2	Two-axle truck
04-06-2018 14:08:16	3	Three-axle truck
04-06-2018 14:08:21	4	Passenger car

## Data Availability

Not applicable.
